# Incremental Value of Plaque Enhancement in Patients with Moderate or Severe Basilar Artery Stenosis: 3.0 T High-Resolution Magnetic Resonance Study

**DOI:** 10.1155/2017/4281629

**Published:** 2017-09-17

**Authors:** Wanqian Wang, Qi Yang, Debiao Li, Zhaoyang Fan, Xiaoming Bi, Xiangying Du, Fang Wu, Ye Wu, Kuncheng Li

**Affiliations:** ^1^Department of Radiology, Xuanwu Hospital, Capital Medical University, Beijing, China; ^2^Biomedical Imaging Research Institute, Cedars-Sinai Medical Center, Los Angeles, CA, USA; ^3^MR R&D, Siemens Healthineers, Los Angeles, CA, USA; ^4^Beijing Key Laboratory of Magnetic Resonance Imaging and Brain Informatics, Beijing, China

## Abstract

**Aim:**

To investigate the clinical relevance of plaque's morphological characteristics and distribution pattern using 3.0 T high-resolution magnetic resonance imaging (HRMRI) in patients with moderate or severe basilar artery (BA) atherosclerosis stenosis.

**Materials and Methods:**

Fifty-seven patients (33 symptomatic patients and 24 asymptomatic patients) were recruited for 3.0 T HRMRI scan; all of them had >50% stenosis on the BA. The intraplaque hemorrhage (IPH), contrast-enhancement pattern, and distribution of BA plaques were compared between the symptomatic and asymptomatic groups. Factors potentially associated with posterior ischemic stroke were calculated by multivariate analyses.

**Results:**

Enhancement of BA plaque was more frequently observed in symptomatic than in asymptomatic patients (27/33, 81.8% versus 11/24, 45.8%; *p* < 0.01). In multivariate regression analysis, plaque enhancement (OR = 7.193; 95% CI: 1.880–27.517; *p* = 0.004) and smoking (OR = 4.402; 95% CI: 2.218–15.909; *p* = 0.024) were found to be independent risk factors of posterior ischemic events in patients with BA stenosis >50%. Plaques were mainly distributed at the ventral site (39.3%) or involved more than two arcs (21.2%) in the symptomatic group but were mainly distributed at left (33.3%) and right (25.0%) sites in the asymptomatic group.

## 1. Introduction

A fifth of all transient ischemic attacks (TIAs) and ischemic strokes are in the territory of the vertebrobasilar circulation. The atherosclerotic plaque of vertebrobasilar artery is the main reason leading to recurrent posterior circulation symptoms [1]. In histopathologic study, basilar artery (BA) atherosclerosis plaque components do not differ qualitatively from coronary plaque [2]; atherosclerosis plaque of BA may have remodeling exactly as in coronary arteries [3]. Some researchers believed that evaluating detailed information of atherosclerotic plaque is more important than measuring arterial lumen stenosis only because atherosclerotic plaque formation and rupture may lead to a hemodynamic change on the vessel walls before the acute ischemic events [4]. Due to the small diameter of intracranial arteries, submillimeter spatial resolution imaging technology is required for visualizing intracranial vessel walls.

Conventional vascular imaging diagnostic approaches such as MR angiography (MRA), digital subtraction angiography (DSA), and CT angiography (CTA) were commonly used to assess extracranial and intracranial artery stenosis in the past few decades. However, these imaging techniques have the limitations that they only show luminal narrowing and occlusion without providing information of the vascular wall.

High-resolution MR imaging (HRMRI) is a fast growing imaging technique for assessing vessel wall characteristics with suppression of arterial blood and cerebrospinal fluid (CSF) signals. With such technique, high-resolution intracranial vessel wall images are usually acquired using 1.5 or 3.0 T MRI scanners [5]. 3.0 T MRI has higher signal-to-noise ratio and spatial resolution than 1.5 T MRI which has more advantages for vascular wall imaging techniques. HRMRI also provides more plaque information than other imaging methods. A few recent studies have confirmed the feasibility of using HRMRI to depict plaque morphology, burden, and distribution [6, 7]. HRMRI can be used for identifying vasculitis and artery dissection [8, 9].

Using HRMRI, researchers found more atherosclerotic lesions on the BA which were not visualized by the conventional MRA method [10]. Assessed by HRMRI, BA plaques had a higher incidence in patients with progressive motor deficits (52%) than in patients without (33%) [11]. Previous studies have shown that positive remodeling lesions were more frequently observed in patients with advanced BA atherosclerosis [12]. In Marquardt et al.'s study, the risk of having posterior TIA and ischemic stroke was found to be significantly higher in patients with >50% vertebrobasilar artery stenosis [13]. However, whether the difference of plaque morphological characteristics and position determines the occurrence of posterior circulation symptoms in patients with moderate or severe BA stenosis is unknown.

The aims of this study were to compare plaque's morphological characteristics and distribution pattern between the symptomatic and asymptomatic groups using 3.0 T HRMRI and to investigate risk factors of posterior ischemic events in patients with BA stenosis > 50%.

## 2. Materials and Methods

### 2.1. Patients

This study was approved by the institutional ethics committee of our hospital. Between June 2014 and July 2016, consecutive patients with confirmed BA stenosis were recruited. The inclusion criteria were as follows: (a) symptomatic patients who had posterior ischemic stroke confirmed by clinical MRI examination within four weeks (acute stroke: 0–7 days; subacute stroke: 7–28 days) and asymptomatic patients who were diagnosed with other diseases without history of cerebrovascular event but had BA stenosis confirmed on image screening as well as patients who had a previous stroke event that occurred outside of the BA territory. All enrolled subjects had moderate (stenosis: 50%–69%) or severe (stenosis: 70%–99%) BA stenosis based on the findings of preceding CTA and/or DSA [14]. The exclusion criteria were as follows: (a) stenosis > 50% in a large extracranial artery based on the findings of preceding CTA and/or DSA, (b) patients with BA occlusion, (c) evidence of cardiac sources of emboli, (d) evidence of nonatherosclerotic intracranial vascular disease, (e) images that cannot be evaluated because of poor quality, and (f) patients having contraindications for MRI and gadolinium-containing contrast agents. The inclusion and exclusion criteria were used in both symptomatic and asymptomatic groups.

### 2.2. Imaging Protocol

All patients signed an informed consent before the HRMRI examinations. All subjects were scanned on a 3.0 T MR scanner (MAGNETOM Verio; Siemens, Erlangen, Germany) equipped with a 32-channel head coil. HRMRI was performed using precontrast and postcontrast 3D T1-weighted imaging-SPACE (3D T1WI-SPACE) sequence [12] and 3D time-of-flight (TOF) MRA sequence. Before acquisition of the contrast-enhanced 3D T1WI-SPACE sequence, 0.2 ml/kg of gadolinium contrast agent (0.1 mg/ml; Magnevist; Schering, Berlin, Germany) was administered to the patient.

HRMRI has good blood suppression properties and high sampling efficiency [15]. Other standard MR imaging protocols included T2-weighted imaging (T2WI), fluid attenuated inversion recovery (FLAIR), and diffusion-weighted imaging (DWI).

The following sequence parameters were selected: 3D T1WI-SPACE MR imaging sequence: TR/TE = 900/15 ms, field of view (FOV) = 170 × 170 mm^2^, slice thickness = 0.53 mm, and voxel size = 0.5 × 0.5 × 0.5 mm^3^; the 3D TOF MRA sequence: TR/TE = 20/3.6 ms, FOV = 220 × 220 mm^2^, slice thickness = 0.7 mm, and voxel size = 0.7 × 0.7 × 0.7 mm^3^.

### 2.3. Image Evaluation

A commercial workstation (syngo.via; Siemens, Erlangen, Germany) was used for MR image analysis by two neuroradiologists; both were blinded to the final diagnosis. Discrepancies between the two neuroradiologists were solved by visual consensus. MR vessel wall images were blinded to clinical information and other MR images. Precontrast and postcontrast 3D T1WI-SPACE images and 3D TOF MRA images were registered and reconstructed in short and long axis views for assessing BA plaque's morphological features.

**Figure 1:**
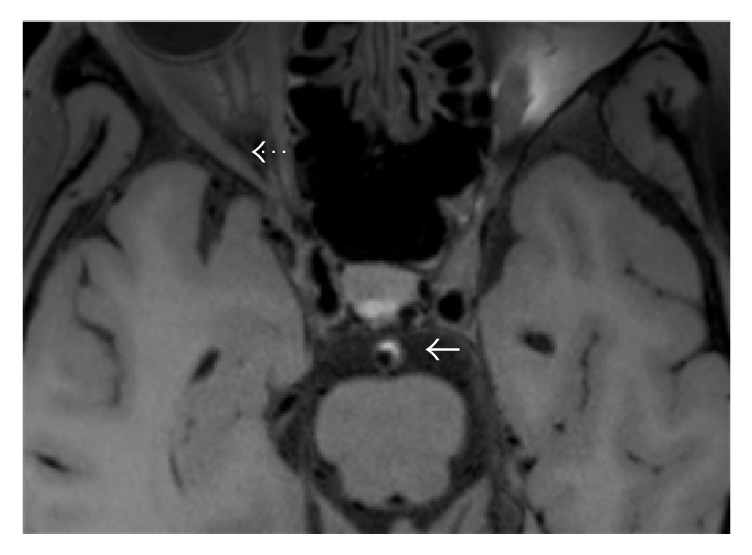
Intraplaque hemorrhage (IPH) was defined as plaque with hyperintensity signal (solid arrow) which was >150% of the extraocular muscle (dotted arrow) on precontrast 3D T1WI-SPACE images.

**Figure 2:**
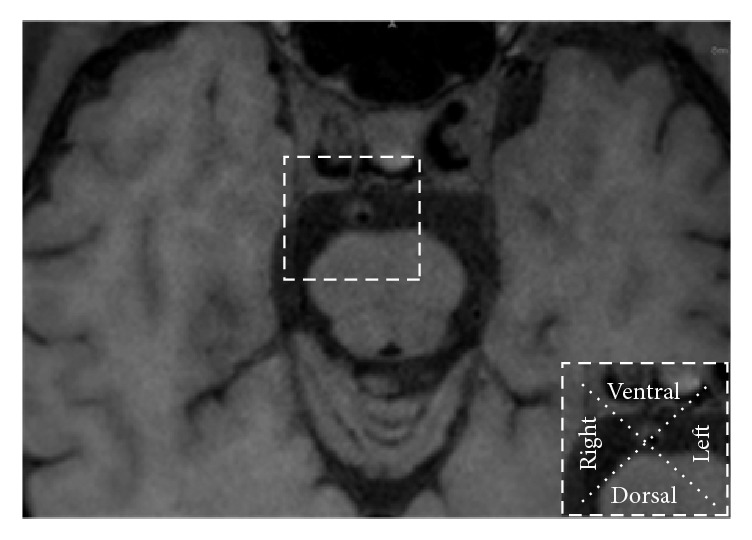
The plaque of BA was evaluated by dividing into four equal arcs (dorsal, ventral, right, and left) on the short axial.

BA stenosis was estimated on 3D T1WI-SPACE images. The stenosis degree was calculated as percent stenosis = [1 − (*D*(stenosis)/*D*(normal))] × 100%. All patients were classified as moderate (stenosis: 50%−69%) or severe (stenosis: 70%−99%) BA stenosis [14]. We recorded plaque distribution at the BA and evaluated thickening and enhancement patterns of the lesion on precontrast and postcontrast 3D T1WI-SPACE images. The plaques at BA were characterized as either eccentric (<50% wall involvement) or concentric (>50% wall involvement). The thickening pattern was scored as focal (defined as a short region or focal point lesion) or diffuse (defined as a lesion over a longer trajectory, e.g., >0.5 cm). Intraplaque hemorrhage (IPH) on BA was defined as the plaque with hyperintensity signal that was >150% of the adjacent muscle (such as extraocular muscle) on precontrast 3D T1WI-SPACE images (Figure 1) [16]. The plaque of BA was evaluated by dividing into four equal arcs (dorsal, ventral, right, and left) on the short axial T1WI-SPACE (Figure 2) [17]. If the plaque extended from one arc to another arc and involved >50% of another arc, it was calculated as ≥2 arcs. Plaque enhancement was compared using precontrast and postcontrast 3D T1WI-SPACE images, where the signal intensity of the plaque was compared with the signal intensity of pituitary. Plaque enhancement was graded using the following scoring system: grade 0, which indicates no enhancement of plaque; grade 1, which indicates that enhancement of plaque was less than the pituitary; and grade 2, which indicates that enhancement of plaque was similar to or greater than the pituitary (Figure 4).

### 2.4. Statistical Analysis

SPSS 19.0 package for Windows (SPSS Inc., Chicago, IL, USA) was used for statistical analysis. We divided the patients into asymptomatic and symptomatic groups. Chi-square tests were used to assess plaque morphological features in both groups. The following variables were analyzed: age, gender, hypertension, hyperlipidemia, diabetes, smoking, severe stenosis, eccentric plaque, focal thickening, enhancement, and IPH. Multivariate logistic analysis with a forward stepwise method was performed including all variables with a probability value < 0.05 in the univariate analysis.

## 3. Results

### 3.1. Patient Characteristics

Fifty-seven consecutive patients (mean age: 59.4 ± 8.1 years, range: 43–78 * *years; 44 males and 13 females) who met the inclusion criteria were finally enrolled and 39 patients were excluded. Mean time interval between onset of clinical symptoms and HRMRI scan was 13.4 days (range from 1 to 28 days). Patients were classified as symptomatic (*n* = 33) and asymptomatic (*n* = 24). Patients' demographic data and cerebrovascular risk factors were illustrated in Table 1. In the symptomatic group, all patients had ischemic strokes (acute stroke, *n* = 18; subacute stroke, *n* = 15).

### 3.2. Plaque's Morphological Features

**Figure 3:**
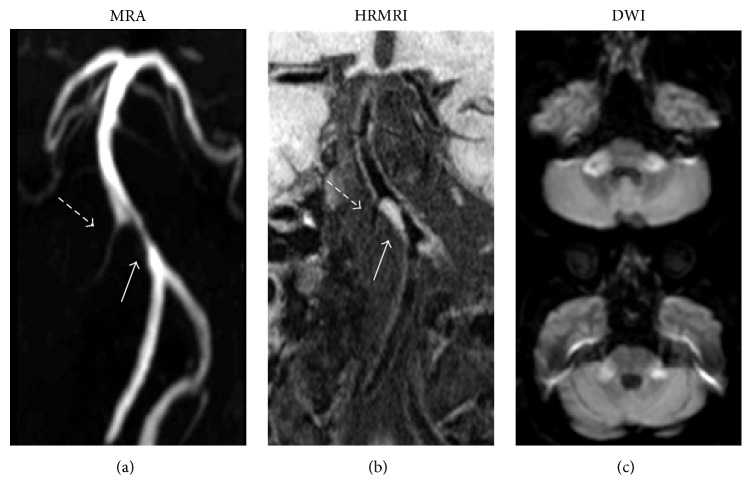
Images from a 65-year-old man with subacute bilateral brachium pontis infarction. (a) MRA shows stenosis of BA (solid arrow) and right anterior inferior cerebellar artery (dashed arrow). (b) T1WI-SPACE images (coronal) show plaque on BA (solid arrow), right anterior inferior cerebellar artery (dashed arrow), and anatomical relationship between orifices of right anterior inferior cerebellar artery and plaque.

**Figure 4:**
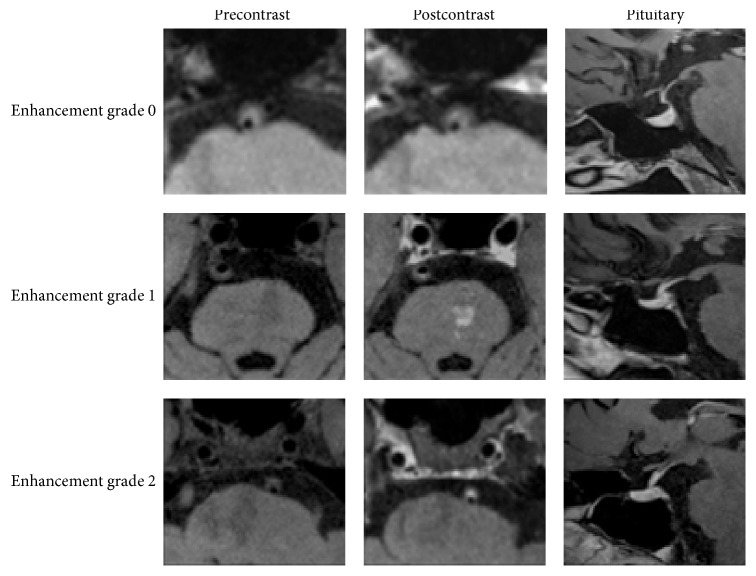
Plaque enhancement was graded by grade 0, grade 1, and grade 2. Grade 0 indicates no enhancement of plaque; grade 1 indicates that enhancement of plaque was less than the pituitary; grade 2 indicates that enhancement of plaque was similar to or greater than the pituitary.

Analyses of plaque's morphological features were illustrated in Table 2 and Figure 3. Enhancement of plaque was observed in 27 (81.8%) symptomatic patients, a percentage which was significantly higher than that (11, 45.8%)) in the asymptomatic group (*p* < 0.01). Other plaque's morphological characteristics did not differ between asymptomatic group and symptomatic group.

### 3.3. Univariate and Multivariate Regression Analysis

In univariate analysis, posterior ischemic events in patients with BA stenosis > 50% were more frequently observed in smokers (*p* = 0.045) and plaque enhancement (*p* = 0.006) compared with patients without ischemic events (Table 3). Other clinical characteristics and HRMRI findings did not differ between both groups. In multivariate regression analysis, plaque enhancement (OR = 7.193; 95% CI: 1.880 to 27.517; *p* = 0.004) and smoking (OR = 4.402; 95% CI: 1.218 to 15.909; *p* = 0.024) were found to be independent risk factors of posterior ischemic events in patients with BA stenosis > 50% (Table 4).

### 3.4. Plaque Enhancement Grade

**Figure 5:**
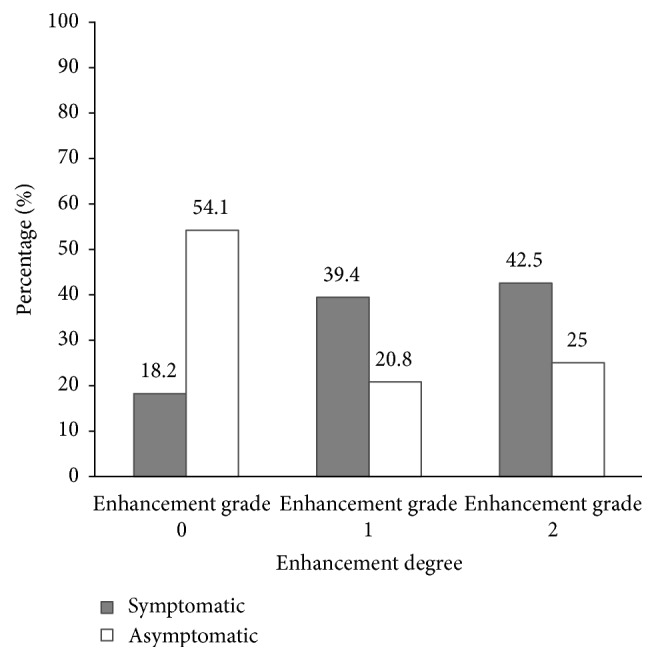
Degree of enhancement in asymptomatic and symptomatic patients.

The enhancement grade of plaque was different in symptomatic group and asymptomatic group (Figure 5). In asymptomatic group, the degree of enhancement was 54.1%, 20.8%, and 25.0%, respectively, for grades 0, 1, and 2, respectively. In symptomatic group, the degree of enhancement was 18.2%, 39.4%, and 42.5% for grades 0, 1, and 2, respectively. Contrast enhancement of plaque was associated with IPH in the symptomatic group (*p* = 0.02; *r* = 0.41) and was not associated with IPH in the asymptomatic group (*p* = 0.78; *r* = 0.06).

### 3.5. Plaque Distribution

The distribution of BA plaques was shown in Table 5. In asymptomatic patients, the plaque's distribution was 20.8% on the ventral wall, 33.3% on the left wall, 8.3% on the dorsal wall, and 25.0% on the right wall, with 12.5% involving more than two arcs. In symptomatic patients, it was 39.3% on the ventral wall, 15.1% on the left wall, 12.1% on the dorsal wall, and 12.1% on the right wall, with 21.2% involving more than two arcs.

## 4. Discussion

In this study, we evaluated plaque's morphology and compared morphological features of symptomatic and asymptomatic patients with BA stenosis > 50%. We demonstrate that (1) contrast enhancement of plaque was significantly high in symptomatic group and that (2) enhancement of plaque was found to be an independent risk factor for posterior ischemic events.

In our current analysis, contrast enhancement of plaque in the symptomatic group was more frequent than that in the asymptomatic group (*p* < 0.01). Our finding was similar to those of previous studies in both the coronary and extracranial carotid arteries [18, 19]. The mechanisms of atherosclerotic plaque enhancement are likely complex and multifactorial. The previous study found that contrast enhancement of plaques in extracranial carotid arteries was associated with vessel wall neovascularization and inflammation [20]. Qiao et al. studied the degree of contrast enhancement in middle cerebral artery (MCA) and showed a strong association between increasing degrees of adventitial enhancement and the recent occurrence of ipsilateral cerebrovascular ischemic events [21]. Furthermore, plaque enhancement was found to be associated with IPH in the symptomatic group (*p* < 0.05) in this study. IPH is typically caused by a rupture in the plaque neovasculature. Contrast enhancement of plaque detected on MRI may be related to endothelial dysfunction present in the diseased intraplaque microvasculature of coronary atherosclerotic vessels [22]. Such process leads to gadolinium accumulation in the perivascular spaces and becomes hyperintense on T1WI MRI sequences.

In multivariate analysis, smoking and plaque enhancement act as independent risk factors for posterior ischemic events when patient's BA stenosis is >50%. Smoking has been proven to be an important risk factor for stroke in many studies over the past decade [23]. In plaque enhancement, the OR value is 7.193, indicating that it could act as an imaging marker to predict patients with higher risk for posterior ischemic events. Interestingly, results from our data also indicated that severe (stenosis: 70%–99%) BA stenosis is not an independent risk factor for posterior ischemic events. This result suggests that simple assessment of BA stenosis has limited value in predicting the risk of ischemic stroke. In Saam et al.'s study, basing decisions on the degree of carotid artery stenosis alone results in misinterpretation of the disease [24]. Thus, identifying imaging markers of BA stenosis may lead to better diagnosis of patients and better decision-making for clinicians.

The main sites of plaque distribution were different in the two groups. In symptomatic group, plaques were commonly found at ventral site and tend to involve more than one arc. This finding is consistent with a previous study, which included 38 posterior circulation symptomatic patients; the BA plaque more likely involved the ventral wall [25]. The authors hypothesized that a part of the orifices of pontine perforators originated from ventral site. In low-grade (<50%) BA stenosis patients, 71.5% of plaques appear to develop at ventral and dorsal walls [26]. However, our results were different from Ravensbergen's postmortem histological study; plaques on BA were more often found at the lateral walls [27]. This discrepancy can be explained by the following: first, the sample size is relatively small; only 17 human cadavers were enrolled in their study; second, the authors did not compare the plaque's distribution between symptomatic and asymptomatic cadavers. The underlying mechanism of localization of atherosclerotic plaques may relate to the complicated flow patterns and low wall shear stress. HRMRI can clearly show the location of plaques; prospective studies are needed to monitor the progression of plaque's enlargement on the vessel wall.

In the present study, T1WI-SPACE sequence was used for evaluating the morphological features of the atherosclerotic plaque on BA. Compared with conventional MRA, T1WI-SPACE sequence provided more detailed information about atherosclerotic stenosis, such as plaque's spatial distribution and plaque's components. The T1WI-SPACE sequence has been widely applied for contrast-enhancement examination to observe the carotid and intracranial arterial plaque [15, 28]. It also provides excellent boundary for vessel wall measurements. From a clinical perspective, T1WI-SPACE sequence can potentially be used to detect the dynamic changes of the atherosclerotic plaque.

This study has several limitations. First, pathological validation was not performed; thus, no pathological evidence was found to explain the mechanism of intracranial plaque enhancement on HRMRI. Second, due to scan time limitations, precontrast and postcontrast 3D T1WI-SPACE images were acquired, but 3D T2WI-SPACE sequence was not included in the imaging protocol. T2WI-SPACE sequence had better contrast-to-noise ratio for lipid core than 3DT1WI-SPACE sequence. IPH has various intensity signals on T2W-SPACE sequence in different periods. Finally, the sample size of our study is relatively small, and larger patient cohorts are needed to validate our findings.

## 5. Conclusion

HRMRI is a promising vessel wall imaging technique for the delineation of BA plaque's morphology and components beyond luminal imaging. Enhancement of plaque may identify plaque's vulnerability and act as a novel imaging marker to predict posterior ischemic events.

## Figures and Tables

**Table 1:** Demographic data and vascular risk factors of patients.

	Asymptomatic (*n* = 24) (%)	Symptomatic (*n* = 33) (%)	*p*
Age (mean ± SD)	59.3 ± 9.3	62.0 ± 8.5	0.434
Male gender	70.8 (17)	81.8 (27)	0.329
Hypertension	66.7 (16)	60.6 (20)	0.640
Diabetes	50.0 (12)	54.5 (18)	0.734
Hyperlipidemia	12.5 (3)	27.3 (9)	0.177
Smoking	33.3 (8)	60.6 (20)	0.042

**Table 2:** Morphology analysis of basilar artery stenosis.

	Asymptomatic (*n* = 24) (%)	Symptomatic (*n* = 33) (%)	*p*
Eccentric plaque	91.7 (22)	78.8 (26)	0.277
Focal thickening	95.8 (23)	84.8 (28)	0.384
IPH	33.3 (8)	42.4 (14)	0.486
Enhancement^*∗*^	45.8 (11)	81.8 (27)	0.004
Severe stenosis	37.5 (9)	33.3 (11)	0.745

IPH: intraplaque hemorrhage. ^*∗*^Enhancement is a combination of grade 2 and grade 3.

**Table 3:** Univariate analysis of posterior ischemic stroke event with BA stenosis > 50%.

Variable	OR	95% CI	*p*
Age	0.930	(0.862, 1.003)	0.060
Male gender	1.853	(0.532, 6.454)	0.333
Hypertension	0.769	(0.256, 2.309)	0.640
Hyperlipidemia	2.625	(0.627, 10.990)	0.187
Diabetes	1.200	(0.418, 3.441)	0.734
Smoking	3.077	(1.025, 9.235)	0.045
Severe stenosis	0.833	(0.278, 2.500)	0.745
Eccentric plaque	0.338	(0.064, 1.795)	0.203
Focal thickening	2.434	(0.027, 2.235)	0.212
Enhancement	5.318	(1.610, 17.563)	0.006
IPH	1.474	(0.493, 4.401)	0.487

OR: odds ratio; CI: confidence interval; IPH: intraplaque hemorrhage.

**Table 4:** Multivariate analysis of posterior ischemic stroke event with BA stenosis > 50%.

Variable	OR	95% CI	*p*
Enhancement	7.193	(1.880, 27.517)	0.004
Smoking	4.402	(1.218, 15.909)	0.024

OR: odds ratio; CI: confidence interval.

**Table 5:** Distribution of basilar artery atherosclerotic plaque.

	Asymptomatic (*n* = 24) (%)	Symptomatic (*n* = 33) (%)
Ventral	20.8 (5)	39.3 (13)
Dorsal	8.3 (2)	12.1 (4)
Left	33.3 (8)	15.1 (5)
Right	25.0 (6)	12.1 (4)
≥2 arcs	12.5 (3)	21.2 (7)
